# Skill Transfer from Laparoscopic Partial Nephrectomy to the Hugo™ RAS System: A Novel Proficiency Score to Assess Surgical Quality during the Learning Curve

**DOI:** 10.3390/jcm13082226

**Published:** 2024-04-11

**Authors:** Francesco Prata, Salvatore Basile, Francesco Tedesco, Alberto Ragusa, Matteo Pira, Andrea Iannuzzi, Marco Fantozzi, Angelo Civitella, Roberto Mario Scarpa, Rocco Papalia

**Affiliations:** Department of Urology, Fondazione Policlinico Universitario Campus Bio-Medico, 00128 Rome, Italy; salvatore.basile@unicampus.it (S.B.); francesco.tedesco@unicampus.it (F.T.); alberto.ragusa@unicampus.it (A.R.); matteo.pira@unicampus.it (M.P.); andrea.iannuzzi@unicampus.it (A.I.); marco.fantozzi@unicampus.it (M.F.); a.civitella@policlinicocampus.it (A.C.); r.scarpa@policlinicocampus.it (R.M.S.); rocco.papalia@policlinicocampus.it (R.P.)

**Keywords:** laparoscopic surgery, robotic surgery, partial nephrectomy, skills transfer, learning curve

## Abstract

**Background/Objectives**: The absence of validated tools to assess the skill transfer from laparoscopy to robotic surgery remains an unsolved issue in the context of robot-assisted partial nephrectomy (RAPN). We aimed to describe and validate a novel proficiency score to critically evaluate the surgical quality of RAPN with the Hugo™ RAS System (Medtronic, Minneapolis, MN, USA). **Methods**: Between October 2022 and September 2023, 27 consecutive patients underwent off-clamp RAPN for localized renal tumors at our institution. To analyze the learning curve (LC), the cohort was chronologically divided into two phases of 6 months each. Proficiency was defined as the achievement of trifecta while maintaining a comparable intraoperative time in the interquartile range of laparoscopic partial nephrectomy performed by the same surgeon. A logistic binary regression model was built to identify predictors of proficiency achievement. **Results**: A proficiency score was achieved in 14 patients (74.1%). At univariable analysis, number of consecutive procedures > 12 (OR 13.7; 95%CI 2.05–21.1, *p* = 0.007), pathological tumor size (OR 0.92; 95%CI 0.89–0.99, *p* = 0.04) and essential blood hypertension (OR 0.16; 95%CI 0.03–0.82, *p* = 0.02) were found to be predictors of proficiency score. At multivariable analysis, after adjusting for potential confounding factors, number of consecutive procedures > 12 (OR 8.1; 95%CI 1.44–14.6, *p* = 0.03) was the only independent predictor of proficiency score achievement. **Conclusions**: Our results showed that the skills of an experienced laparoscopic surgeon are transferrable to the novel Hugo™ RAS System in the context of nephron-sparing surgery. Improved surgical quality may be expected after completing the first 12 consecutive procedures.

## 1. Introduction

The adoption of robotic platforms has witnessed a notable surge in recent years owing to its distinct advantages in renal tumor resection and parenchyma preservation [1,2]. This provided high standards of intraoperative outcomes and improved postoperative recovery in the context of robotic-assisted partial nephrectomy (RAPN) [3].

The primary objective of RAPN is to achieve negative surgical margins and the absence of local recurrence, serving as surrogates for oncological quality, while guaranteeing renal function preservation in terms of serum creatinine (sCr) and estimated glomerular filtration rate (eGFR). Recently, proficient surgeons from referral centers worldwide have broadened the indications for nephron-sparing surgery (NSS) to include even the most intricate and challenging surgical scenarios, such as totally endophytic tumors and high nephrometry score masses [4,5,6]. Nevertheless, positive surgical margins and adverse pathology may increase the risk of local recurrence and the necessity for subsequent radical treatment, compromising long-term renal function and exposing patients to a higher likelihood of chronic kidney disease and related major cardiovascular events [7,8,9].

Novice surgeons attempting complex renal surgery may encounter a steep learning curve (LC) during their learning process, limiting their proficiency and confidence. The LC embodies the trajectory of skill acquisition and comprehension of a novel surgical technique [10]. A thorough understanding of surgeons’ LC may be instrumental in developing effective and standardized training programs that ensure the attainment of baseline expertise without compromising patient safety during the learning phase [11]. 

The achievement of proficiency during the RAPN LC is deeply influenced by previous surgical exposure, with an emphasis on achieving good oncological quality as the primary goal in RAPN training [12]. Evaluating the RAPN LC is crucial in ensuring patient safety and optimizing surgical outcomes during the learning phase [13]. However, in the setting of complex surgical scenarios, the absence of validated tools to assess the transfer of skills from laparoscopy to robotic surgery remains a pressing concern [14]. On one hand, various studies have investigated LC of RAPN; on the other hand, only a few have thoroughly examined the transferability of skills from laparoscopy to robot-assisted surgery and between different robotic platforms [15,16]. 

The granularity characterizing the aforementioned studies limits the ability to establish standardized training protocols that encompass the nuances of transitioning from laparoscopy to robotic-assisted techniques, hindering the optimization of surgical proficiency and patient outcomes across different surgical modalities. Therefore, there is a critical need for further research to elucidate the factors influencing skill transferability and to develop comprehensive training programs that address the intricacies of transitioning between different surgical platforms. Existing assessment methods often vary in their scope and applicability, ranging from subjective evaluations by experienced surgeons to objective metrics such as operative time, blood loss and complication rates. While these methods offer valuable insights into surgical proficiency, they also possess inherent limitations. Subjective evaluations may be influenced by individual biases, while objective metrics may fail to capture the nuanced skill set required for complex surgical procedures. Additionally, the transferability of proficiency scores across different surgical settings and levels of experience remains a challenge. By addressing these limitations and proposing a novel proficiency score tailored specifically to robotic-assisted partial nephrectomy with the Hugo™ RAS System, our study seeks to fill a critical gap in the literature and provide a standardized framework for evaluating surgical proficiency in this context. Therefore, the present study endeavors to assess the LC associated with RAPN and compare perioperative outcomes across different phases of the LC.

## 2. Materials and Methods

### 2.1. Data Collection

Between October 2022 and September 2023, 27 consecutive patients underwent RAPN for localized renal tumors at our Institution. Baseline characteristics including demographic features, such as gender, age, American Society of Anesthesiologists physical status score (ASA score), body mass index (BMI), Charlson Comorbidity Index, R.E.N.A.L. nephrometry score, clinical tumor size (defined as the maximum diameter of the tumor assessed via CT scan) and comorbidities such as diabetes and hypertension (defined as systolic blood pressure consistently above 140 mmHg or diastolic blood pressure consistently above 90 mmHg) were recorded. Preoperative assessment included estimated glomerular filtration rate (eGFR) and hemoglobin (Hb) levels. Perioperative parameters consisted of operative time (OT), complications graded according to the Clavien–Dindo classification [17], estimated blood loss (EBL), postoperative length of stay (PLOS), postoperative serum creatinine (sCr), eGFR and Hb levels. Pathological outcomes, including histopathology and surgical margin status, were also collected. The eGFR, with normal values typically ranging from 90 to 120 mL/min/1.73 m^2^, was calculated using the Chronic Kidney Disease Epidemiology Collaboration (CKD-EPI) formula. Trifecta achievement was defined by the fulfilment of three criteria: negative surgical margin, decline of eGFR  <  10% and Clavien–Dindo grade < 2 complications. OT was measured from the initial skin incision to wound closure completion.

### 2.2. Surgical Technique

The surgical setup and technique for off-clamp partial nephrectomy using the Hugo™ RAS System have been previously described [18]. Following the administration of general anesthesia and the placement of a transurethral bladder catheter, patients were positioned in an extended flank position with a moderate flexion of approximately 45 degrees. This positioning strategy aimed to optimize the operating space between the ipsilateral iliac spine and the margin of the ribs, facilitating surgical access and maneuverability [19]. The positioning of the bedside assistant, whether standing or seated, was determined based on patient anatomy, surgical bed height and robotic arm docking and tilt angles. The configuration of the Hugo™ RAS trocars involved four robotic arms, including an 11 mm optic port and three 8 mm robotic instruments, with provision for a single laparoscopic trocar for the bedside assistant, which is located on the side of the patient, limiting its “active” participation during crucial steps of RAPN. In both cases of three- or four-arm setting, our surgical setup provides two 12 mm laparoscopic trocars for the bedside assistant. This configuration allowed the contextual usage of two suction devices from the bed assistant for simultaneous irrigation on the resection bed, providing the first surgeon a clear-cut distinction between tumor capsule and benign renal parenchyma during enucleation. Pneumoperitoneum was established using the AirSeal™ system (SurgiQuest, Milford, CT, USA), maintaining standard intra-abdominal pressure of 12 mmHg during the procedure and raising it to 15 mmHg for enucleation. Arm carts and energy tower placement was optimized to ensure efficient workflow and prevent collisions between robotic arms and between robotic arms and the bedside assistant. Surgical personnel, including the primary surgeon, bedside assistants and scrub nurses, underwent comprehensive technical training on the Hugo™ RAS System. In all cases, a transperitoneal route was adopted. Once retroperitoneum was reached, Gerota’s fascia was incised and tumor margins carefully isolated. Incision of renal parenchyma was performed through monopolar robotic shears. Mass enucleation was performed without clamping the main renal artery, with careful management of bleeding using double suction and irrigation. Renorrhaphy was performed with a sliding clips technique using hemostasis, which was achieved using Monocryl 2-0 stitches (Johnson & Johnson, New Brunswick, NJ, USA). Hemostasis was perfectioned through the application of hemostatic agents on the resection bed. Finally, a single drain was inserted and Gerota’s fascia was closed using either Monocryl 2-0 single running suture or Hem-o-lok clips.

### 2.3. Postoperative Management

Key postoperative priorities included closely monitoring vital signs and assessing the contents and output of both the Foley catheter and drainage tube. On the first and second postoperative day, laboratory tests were performed to assess Hb levels, eGFR and sCr. The Foley catheter was removed on first postoperative day and patients were mobilized, while the drain was removed between second and third postoperative day before the patient’s discharge. Postoperative complications were collected according to the Clavien–Dindo classification [17].

### 2.4. Endpoint, Outcome and Statistical Analysis

The primary endpoint of the study was to describe and validate a novel proficiency score to critically evaluate the surgical quality of RAPN with the Hugo™ RAS System. The secondary endpoint was to analyze the LC, splitting chronologically the cohort into two phases of 6 months each: phase I with 12 patients; phase II with the subsequent 15 patients. This division was implemented to maintain homogeneity in terms of both temporal distribution and sample size across the two phases. Proficiency was defined as the achievement of trifecta (negative surgical margin status, no Clavien–Dindo grade ≥ 3 complications and eGFR decline ≤ 30%) while maintaining a comparable intraoperative time in the interquartile range of LPN performed by the same surgeon. The concept of trifecta in partial nephrectomy, comprising negative surgical margin status, minimal perioperative complications and preservation of renal function, has been extensively studied and validated in the literature. Several studies have demonstrated the importance of trifecta achievement as a predictor of favorable surgical outcomes and long-term renal function in patients undergoing partial nephrectomy for renal tumors [20,21]. Notably, ischemia time was not included in our analysis as the procedures were performed off-clamp.

Continuous variables were presented as median and interquartile ranges (IQR), while frequencies and percentages (%) were used to report categorical data. The latter were compared using the χ^2^ test or Fisher’s exact test, while continuous variables were analyzed using the Mann–Whitney U test. Finally, univariate and multivariate logistic regression models were employed in our quest to identify independent predictors of proficiency score.

A *p*-value < 0.05 was considered statistically significant. Statistical analyses were conducted using STATA (StataCorp.2021. Stata Statistical Software: Release 17, StataCorp LLC, College Station, TX, USA).

## 3. Results

### 3.1. Patient Demographic and Preoperative Outcomes

From October 2022 to September 2023, a total of 27 consecutive patients underwent RAPN for localized renal tumors at our institution. Demographic features are summarized in Table 1.

Five patients (18.5%) were female, with a median age of 68 years (IQR, 57–73) and BMI of 27.4 kg/m^2^ (IQR, 25.9–31.2). The median R.E.N.A.L. nephrometry score was 7 (IQR 5–8). A slight preponderance of right-sided renal masses was observed in our cohort (59.3%). Approximately 51.8% of patients required antihypertensive therapy, while only two (7.4%) patients were diagnosed with diabetes. Most patients displayed ASA score II (63%), along with a median Charlson Comorbidity Index of 4 (IQR, 3–5). The median clinical tumor size, as determined by preoperative contrast-enhanced CT scan, was 34 mm (IQR, 26–45), predominantly at a clinical stage of T1a in 21 patients (77.8%). Notably, four patients (11.1%) of the whole cohort displayed T2 renal masses. Regarding preoperative renal function, a median serum creatinine level of 0.93 mg/dL (IQR, 0.81–1.09) and eGFR of 77.5 mL/min/1.73 m^2^ (IQR, 64.2–92.3) were reported. Preoperative Hb levels were 14.7 g/dL (12.3–15.4).

### 3.2. Perioperative Outcomes

Perioperative data are displayed in Table 2.

Median operative time and estimated blood loss were 91 min (IQR, 50–149) and 150 mL (IQR, 50–450), respectively. Among the cohort, three patients (11%) exhibited Clavien–Dindo grade >2 complications (11.3%, two minor and one major). Hemoglobin levels and a median sCr at discharge were 11.2 g/dL (IQR, 9.1–12.3) and 0.93 mg/dL (IQR, 0.82–1.13), respectively, the result being comparable to preoperative values. The average length of hospital stay (LOS) was 3 days (IQR 3–4). Remarkably, only one patient exhibited a positive surgical margin (3.7%), and malignant pathology was confirmed in 21 cases (77.8%), with a prevalence of clear cell renal cell carcinoma (RCC) histology (51.9%). The final pathological staging revealed that, out of the total cohort, 20 patients (74.1%) were classified as pT1a, 3 patients (11.1%) as pT1b, 2 patients (7.4%) as pT2a, 1 patient (3.7%) as pT2b and 1 patient (3.7%) as pT3a. At a median follow-up of 12 months (IQR, 8–15), no patients displayed local or distant recurrence at conventional contrast-enhanced CT scan imaging and no renal function deterioration was observed in comparison to perioperative values (Table 2). Overall, proficiency score was achieved in 20 patients (74.1%).

On univariable analysis, number of consecutive procedures > 12 (OR 13.7; 95%CI 2.05–21.1, *p* = 0.007), pathological tumor size (OR 0.92; 95%CI 0.89–0.99, *p* = 0.04) and essential blood hypertension (OR 0.16; 95%CI 0.03–0.82, *p* = 0.02) were shown to be predictors of proficiency score (Table 3). Multivariable analysis showed that, after adjusting for potential confounding factors, number of consecutive procedures > 12 (OR 8.1; 95%CI 1.44–14.6, *p* = 0.03) was the only independent predictor of proficiency score achievement.

## 4. Discussion

RCC management has evolved significantly over the years, offering a spectrum of treatment options tailored to individual patient needs. Among these options, partial nephrectomy has emerged as the preferred approach for managing localized T1-stage tumors due to its preservation of renal function [12,22,23]. Since the introduction of a robotic platform in the context of NSS, surgical precision and surgical outcomes have experienced significant enhancements, leading to a growing acceptance and utilization in clinical settings [24,25,26].

In recent years, several innovative robotic platforms have entered the market, with Hugo RAS™ System by Medtronic emerging as particularly promising [27]. Renowned for its ergonomic design, this system offers enhanced flexibility and the capability to customize surgical settings to a remarkable degree. In contrast to Medtronic’s proposed setup, we opted for a singular configuration. This decision was made to afford greater freedom to the surgical assistant and minimize potential conflicts between instruments [18,19].

The transition from conventional laparoscopic techniques to robotic-assisted surgery represents a paradigm shift in the field of urology. While laparoscopy has long been regarded as the gold standard for minimally invasive nephron-sparing surgery (NSS), the introduction of robotic technology has brought about significant improvements in procedural efficiency and patient outcomes. By leveraging the benefits of robotic-assisted techniques, surgeons can achieve superior results while minimizing the risks associated with open surgery. One of the key challenges in adopting new surgical technologies is ensuring proficiency among surgical teams. Proficiency in RAPN requires not only technical skill but also an understanding of the unique capabilities and limitations of robotic platforms. As such, there is a growing need for standardized methods of evaluating surgical quality and performance in the context of robotic-assisted procedures.

To address this need, our study focused on skill transfer from a conventional laparoscopic approach to RAPN, aiming to evaluate its LC and perioperative outcomes while proposing a novel proficiency score for assessing surgical quality with the Hugo™ RAS System. Our approach was inspired by previous studies, such as the work by Kim et al. [28], who introduced a proficiency score for laparoscopic radical prostatectomy. By incorporating key metrics such as operative time, blood loss, and trifecta achievement, this score provides a comprehensive framework for evaluating surgeon performance and optimizing patient outcomes. Furthermore, our study sought to identify predictors of proficiency, such as the number of consecutive procedures and tumor size, to inform future training programs and enhance skill acquisition among surgical teams. In this study we aim to envision the generalizability of the proficiency score across different surgical settings and varying levels of surgeon experience by proposing a comprehensive framework that assesses surgical quality and performance in the context of robotic-assisted partial nephrectomy (RAPN) with the Hugo™ RAS System. The study cohort comprised 27 consecutive patients undergoing RAPN, primarily for localized renal tumors. The division of our study cohort into two phases over a 6-month period was designed to maintain homogeneity in terms of temporal and numerical factors. This approach allowed us to analyze the learning curve of RAPN with the Hugo™ RAS System and identify trends in proficiency and perioperative outcomes over time. By examining the progression of surgical skill acquisition, we aimed to provide valuable insights into the learning process and guide the development of training protocols for novice robotic surgeons. The study cohort comprised 27 consecutive patients undergoing RAPN, primarily for localized renal tumors. Patient demographics revealed a typical RCC population, with a mean age of 68 years and a predominance of hypertensive individuals (OR 0.16; 95%CI 0.03–0.82, *p* = 0.02). These findings resonate with RCC epidemiology and highlight the importance of addressing comorbidities in surgical decision making. Preoperative hypertension, defined as blood pressure greater than 140/90 mmHg, was present in 51.8% of patients, reflecting the prevalence of this condition in the RCC patient population and its potential impact on surgical outcomes. Perioperative outcomes demonstrated favorable results, with a proficiency score achievement of 74.1%. This emphasizes the feasibility and efficacy of RAPN in experienced hands. Complication rates were low, aligning with contemporary literature advocating for the safety of robotic-assisted techniques. Furthermore, trifecta achievement was reached in a significant proportion of cases (74%). These outcomes reaffirm the oncological and functional benefits associated with partial nephrectomy.

Similarly to the study conducted by Mottrie et al. [29], which observed optimization in surgical proficiency after 20–30 consecutive procedures, our study independently demonstrates a similar trend in the learning curve trajectory with the attainment of proficiency using our novel proficiency score. The LC analysis revealed interesting insights into skill acquisition and procedural refinement. The study identified the number of consecutive procedures (>12) as an independent predictor of proficiency score achievement (OR 8.1; 95%CI 1.44–14.6, *p* = 0.03). This underlines the importance of surgical exposure in mastering RAPN, reflecting the need for structured training programs and mentorship to expedite the learning process. Additionally, a median pathological tumor size of 30 mm emerged as a predictor of proficiency score (OR 0.92; 95%CI 0.89–0.99, *p* = 0.04) reflecting the complexity of cases encountered during the learning phase.

The proposed proficiency score represents a novel tool aimed at standardizing surgical assessment in RAPN with the Hugo™ RAS System. Its validation underscores the study’s contribution to refining surgical assessment methodologies and optimizing patient outcomes in complex surgical scenarios.

Despite the promising findings, several limitations need to be considered. The study’s retrospective design and single-surgeon experience may limit generalizability to broader surgical cohorts. Additionally, the small sample size necessitates cautious interpretation of results, highlighting the need for larger-scale studies to validate findings and establish robust learning curve parameters.

Notwithstanding these limitations, our study focused on the LC of a single surgeon with previous extensive expertise in laparoscopic techniques, aiming to unravel the progression of surgical skill acquisition and scrutinize perioperative outcomes. By delving into the learning process and performance of RAPN, this study offered invaluable insights, ultimately enhancing patient care and surgical proficiency. Through meticulous examination and validation of this proficiency score, we aimed to contribute to the refinement of surgical assessment methodologies and ultimately optimize patient outcomes in RAPN procedures.

## 5. Conclusions

Our results showed that the skills of an experienced laparoscopic surgeon are transferrable to the novel Hugo™ RAS System in the context of NSS. RAPN with Hugo™ RAS seemed to be safe and efficient in terms of perioperative and functional outcomes since the beginning of the LC. The proposed proficiency score offers a valuable tool for assessing surgical quality and enhancing procedural standardization. Improved surgical quality may be expected after completing the first 12 consecutive procedures. A surgeon with previous exposure to laparoscopy should consider this novel threshold as a baseline standard to attain a satisfactory level of surgical efficacy. In light of our findings, it is crucial to offer preliminary recommendations for a broader audience of healthcare practitioners. Our analysis underscores the pivotal role of surgical proficiency in ensuring favorable outcomes in robotic kidney tumor operations. Therefore, we suggest prioritizing ongoing development and refinement of these skills. While recognizing the importance of other factors such as tumor size, blood pressure and glomerular filtration rate, surgical proficiency remains a key determinant of surgical success. By integrating these recommendations into clinical practice, healthcare providers can contribute to improved clinical outcomes in renal surgery.

## Figures and Tables

**Table 1:** Baseline and demographic data.

Variable	Cohort (n = 27)
Age (n, median, IQR)	68 (57–73)
Gender (n, %) - Male - Female	- 22 (81.5%) - 5 (18.5%)
BMI (kg/m^2^, median, IQR)	27.4 (25.9–31.2)
ASA score (n, %) - I - II - III - IV	- 1 (3.7%) - 17 (63%) - 9 (33.3%) - 0
Charlson Comorbidity Index (median, IQR)	4 (3–5)
Diabetes (n, %)	2 (7.4%)
Hypertension (n, %)	14 (51.8%)
Preoperative Hemoglobin (g/dL, median, IQR)	14.7 (12.3–15.4)
Preoperative Creatinine (mg/dL, median, IQR)	0.93 (0.81–1.09)
Preoperative eGFR (mL/min/1.73 m^2^, median, IQR)	77.5 (64.2–92.3)
Clinical Tumor Size (mm, median, IQR)	34 (26–45)
cT (n, %) - T1a - T1b - T2a - T2b	- 21 (77.8%) - 3 (11.1%) - 2 (7.4%) - 2 (3.7%)
Side (n, %) - Right - Left Bilateral	- 16 (59.3%) - 11 (40.7%) 0
R.E.N.A.L. score (median, IQR)	7 (5–8)

**Table 2:** Perioperative data.

Variable	Cohort (n = 27)
Operative Time (min, median, IQR)	91 (50–149)
Estimated blood loss (ml, median, IQR)	150 (50–450)
Clavien–Dindo complications (n,%) - I - II - IIIa	- 0 - 2 (7.4%) - 1 (3.7%)
Length of Stay (days, median, IQR)	3 (3–4)
Hemoglobin at discharge (g/dL, median, IQR)	11.2 (9.1–12.3)
Creatinine at discharge (mg/dL, median, IQR)	0.93 (0.82–1.13)
eGFR at discharge (mL/min/1.73 m^2^, median, IQR)	74.9 (63–92.1)
Readmission (n, %)	0 (0%)
Pathological Size (mm, median, IQR)	30 (20–43)
Pathology (n, %) - Benign - Malignan	- 6 (22.2%) - 21 (77.8%)
Histology subtype (n, %) - Angiomyolipoma - Oncocytoma - Clear Cell RCC - Papillary RCC - Cromophobe - Other	- 2 (7.4%) - 4 (14.8%) - 14 (51.9%) - 5 (18.5%) - 2 (7.4%) - 0 (0%)
Positive Margins (n, %)	1 (3.7%)
pT Stage (n, %) - 1a - 1b - 2a - 2b - 3a	- 20 (74.1%) - 3 (11.1%) - 2 (7.4%) - 1 (3.7%) - 1 (3.7%)
Creatinine at last follow-up (mg/dL, median, IQR)	0.99 (0.85–1-14)
eGFR at last follow-up (mL/min/1.73 m^2^, median, IQR)	79.3 (58.3–87.8)
Trifecta achievement rate (n, %)	20 (74.1%)
Proficiency Score (n, %)	20 (74.1%)

**Table 3:** Univariable and multivariable logistic regression analysis evaluating predicting factors of proficiency score achievement during RAPN learning curve.

Variable	Univariate Analysis				Multivariate Analysis			
	Odds Ratio	95.0% CI			Odds Ratio	95.0% CI		
		Inferior	Superior	*p*-Value		Inferior	Superior	*p*-Value
**Age**	1.03	0.96	1.10	0.37	-	-	-	-
**Gender**	0.61	0.08	4.37	0.62	-	-	-	-
**BMI**	0.89	0.73	1.08	0.24	-	-	-	-
**ASA score**	0.33	0.07	1.55	0.16	-	-	-	-
**Side**	0.58	0.19	1.76	0.34	-	-	-	-
**Diabetes**	1.05	0.66	1.68	0.81	-	-	-	-
**Hypertension**	**0.16**	**0.03**	**0.82**	**0.02**	0.11	0.01	2.07	0.14
**Preoperative Hb**	0.66	0.38	1.16	0.15	-	-	-	-
**Preoperative eGFR**	0.98	0.92	1.02	0.53	-	-	-	-
**R.E.N.A.L. score**	0.71	0.56	0.99	0.09	-	-	-	-
**Clinical tumor size**	0.96	0.92	1.01	0.13	-	-	-	-
**Docking time**	1.1	0.90	1.35	0.32	-	-	-	-
**Estimated Blood Loss**	0.99	0.98	1.00	0.11	-	-	-	-
**Length of stay**	0.62	0.08	11.2	0.95	-	-	-	-
**Pathological tumor size**	**0.92**	**0.89**	**0.99**	**0.04**	**0.91**	**0.82**	**1.01**	**0.09**
**Number of consecutive procedures (>12 vs. <12)**	**13.7**	**2.05**	**21.1**	**0.007**	**8.1**	**1.44**	**14.6**	**0.03**

## Data Availability

The data presented in this study are available on request from the corresponding author.
